# Editorial: Regulation Mechanism of Adipose-Derived Stem Cells in Differentiation and Translation

**DOI:** 10.3389/fphys.2022.852275

**Published:** 2022-02-25

**Authors:** Jun Fan, Jingxing Dai, Feng Lu, Yuanyuan Zhang

**Affiliations:** ^1^Department of Tissue Engineering, School of Intelligent Medicine, China Medical University, Shenyang, China; ^2^Guangdong Provincial Key Laboratory of Medical Biomechanics and Department of Anatomy, School of Basic Medical Science, Southern Medical University, Guangzhou, China; ^3^Department of Plastic and Cosmetic Surgery, Nanfang Hospital, Southern Medical University, Guangzhou, China; ^4^Wake Forest Institute for Regenerative Medicine, Wake Forest School of Medicine, Winston-Salem, NC, United States

**Keywords:** adipose-derived stem cells, differentiation, regulation mechanism, translation medicine, regenerative medicine

Adipose-derived stem cells (ASCs) are a population of mesenchymal stem cells with the multipotent capability in adipose tissue, which can be easily harvested during liposuction or resection of adipose tissue. A series of studies have demonstrated that ASCs can differentiate into many different cell types including osteocytes, chondrocytes, vascular endothelial cells, and adipocytes (Wang et al., 2020). ASCs have received considerable attention over the past decades, mainly because of their therapeutic potential in different areas of applied science. Cell-based therapies using ASCs are increasingly being reported. Furthermore, a better understanding of adipogenesis of ASCs may also promote developing methods for the treatment and prevention of obesity. Therefore, exploring the molecular mechanisms of multipotency, proliferation, migration/homing, and immunosuppression in ASCs are critical for understanding the therapeutic ability of ASCs in clinic, which will lay a foundation for further application of ASCs in tissue regeneration and translation. In the Research Topic, the original research articles and reviews focused on the regulation mechanism of ASCs in differentiation as well as their translational application.

ASCs reside in a special three-dimensional (3D) microenvironment (niche), which plays important role on the maintenance of stemness and tissue regeneration (Milan et al., 2021). The stem cell niche can be influenced by surrounding cells, signaling molecules, matrix architecture, physical forces, oxygen tension, and other environmental factors. Hypoxia plays a critical role in the maintenance of stem cell characteristics. However, the impact of hypoxia on ASC viability and function is still largely unknown (Zhao et al., 2020). Hofmann et al. investigated the effect of migration inhibitory factor (MIF) on ASCs from healthy donor site and chronic wounds under hypoxia. Macrophage migration inhibitory factor MIF as a multifunctional chemokine-like inflammatory cytokine plays an essential role in various acute and chronic inflammatory diseases, autoimmunity, and cardiovascular diseases. They demonstrated that hypoxia did not induce ASCs viability, but showed an obvious up-regulation of MIF secretion in ASCs from chronic non-healing wounds. However, ASCs from healthy donor sites did not increase MIF secretion upon hypoxia. The above results indicated that MIF level in ASCs was increased under the inflammatory conduction. In the following experiments, they found that MIF was involved in the interaction of CD74 (MIF receptor) and downstream activation of ERK and AKT (mediating survival and apoptosis), but did not alter ASCs viability. However, MIF reduced IL-1β secretion. These results indicated that MIF had an impact on the wound environment by modulating inflammatory factors IL-1β. The other cellular functions of MIF in ASCs, such as differentiation or migration should be explored further in larger clinical sample study.

ASCs treatment is a promising therapeutic method in autoimmune and inflammatory diseases (Trzyna and Banaś-Zabczyk, 2021). The biology of ASCs in rheumatic diseases is largely unknown. Recent work identified the basic characterization of ASCs from the rheumatic disease patients, such as systemic lupus erythematosus (SLE), systemic sclerosis (SSc), and ankylosing spondylitis, and compared their phenotype and secretory potential with the ASCs from healthy donors (Kuca-Warnawin et al., 2019). The Original Research article by Warnawin et al. explored the therapeutic effect of ASCs from SLE and SSc patients. T lymphocytes activation plays a fundamental role in the pathogenesis of rheumatic diseases. The experiment demonstrated that ASCs significantly decreased the number of proliferating CD4+ and CD8+ T-cells. SLE/ASCs and SSc/ASCs, like healthy donors (HD/ASCs) could significantly inhibit the expansion of allogeneic CD4+ and CD8+ T-cells. All ASCs acted mainly via soluble factors by regulating kynurenine pathway. In this study, they found that the classical TGFβ was not involved in contributing the ASCs anti-proliferative capabilities. Based on their work, they concluded that SLE/ASCs and SSc/ASCs as ASCs of healthy donors might also be used for therapeutic translation in rheumatic disease patients. This article proved that autologous ASCs in SLE and SSc patients had similar immunosuppressive properties to ASCs of healthy donors.

ASCs could promote tumor progression through their interactions with breast cancer cells and immune cells. On the other side, ASCs were used in breast reconstruction and tissue engineering. In the review article, Brock et al. reviewed the role of ASCs and adipocytes in normal physiology and breast cancer, particularly in obesity. They also evaluated the oncological safety of ASCs for reconstructive surgery based on the publications. This review emphasized that adipocytes and ASCs were good candidates for cancer therapeutics and regenerative medicine.

During the stem cell differentiation process, besides the changes in the cell expression profile, the extracellular matrix (ECM) environment also plays the significant roles in this process. Matrix metalloproteinases (MMPs) are essential for extracellular matrix remodeling, which are suggested to function in adipose tissue growth such as supporting the differentiation of preadipocytes. Recently, a regulatory role of MMP-3 has been suggested in adipogenesis. Wan et al. reviewed the growing knowledge of MMP-3 involvement in regulating cell differentiation and disease progression. Especially, MMP-3 in the development of osteoarthritis was discussed. Another study also found that transplantation of MMP-3 modified ASCs obviously promoted wound healing and reduced scar formation in rabbits (Rong et al., 2020).

In conclusion, the current Research Topic highlighted the essential role of ASCs and the related signal mechanism in the process of differentiation and translation. Our increasing understanding of regulatory frameworks of ASCs features will accelerate the development of novel strategies for clinical applications of ASCs.

## Author Contributions

JF, JD, FL, and YZ: conceptualization and draft manuscript preparation. All authors listed have made a substantial, direct, and intellectual contribution to the work and approved it for publication.

## Funding

This work was supported by the National Natural Science Foundation, China [no. 81571919] and Liaoning Revitalization Talents Program, China [no. XLYC1907124].

## Conflict of Interest

The authors declare that the research was conducted in the absence of any commercial or financial relationships that could be construed as a potential conflict of interest.

## Publisher's Note

All claims expressed in this article are solely those of the authors and do not necessarily represent those of their affiliated organizations, or those of the publisher, the editors and the reviewers. Any product that may be evaluated in this article, or claim that may be made by its manufacturer, is not guaranteed or endorsed by the publisher.
